# Low Zoonotic Pathogen Burden in Free-Roaming Cats Revealed by 18S rRNA Metabarcoding: A Baseline Study from an Insular Natura 2000 Site in Spain

**DOI:** 10.3390/ani16030431

**Published:** 2026-01-29

**Authors:** María del Mar Travieso-Aja, Luis Alberto Henríquez-Hernández, Elisa Hernández-Álvarez, Javier Quinteiro-Vázquez, Nieves E. González-Henríquez, Martina Cecchetti, Octavio P. Luzardo

**Affiliations:** 1 Research Institute of Biomedical and Health Sciences (IUIBS), University of Las Palmas de Gran Canaria, Paseo Blas Cabrera “Físico” s/n, 35016 Las Palmas de Gran Canaria, Spain; marimar.travieso@ulpgc.es (M.d.M.T.-A.); luis.henriquez@ulpgc.es (L.A.H.-H.); elisa.hernandez@alu.ulpgc.es (E.H.-Á.); mc703@exeter.ac.uk (M.C.); 2 Spanish Biomedical Research Center in Physiopathology of Obesity and Nutrition (CIBERObn), Avenida Monforte de Lemos, 5, 28029 Madrid, Spain; 3 Universidad Fernando Pessoa Canarias, 35450 Guía, Spain; 4 Molecular Systematics Laborator (SISMOL), Aquatic One Health Research Center (iARCUS), University of Santiago de Compostela, Avda. Lope Gómez de Marzoa s/n, 15782 Santiago de Compostela, A Coruña, Spain; javier.quinteiro@usc.es; 5 Molecular Biodiversity Laboratory (BIOMOL), Escuela Taller Jaime O’Shanahan, University of Las Palmas de Gran Canaria, 35017 Las Palmas de Gran Canaria, Spain; nieves.gonzalez@ulpgc.es

**Keywords:** free-roaming cats, community cats, Oxford Nanopore, zoonoses, One Health, *Toxoplasma gondii*, *Dipylidium caninum*, Natura 2000

## Abstract

Free-roaming cats may contribute to zoonotic risk via parasites and other eukaryotic taxa, yet surveillance in protected island settings is limited and conventional coprology can miss low-intensity or degraded signals. We conducted a cross-sectional 18S rRNA metabarcoding survey to establish a baseline profile of potentially pathogenic eukaryotes in community cats from La Graciosa (Natura 2000, Canary Islands, Spain) prior to large-scale antiparasitic interventions. We analysed 152 faecal samples, including fresh samples collected during a high-throughput TNR campaign (*n* = 37) and dry environmental deposits (*n* = 115). Host amplification was reduced using a feline 18S blocking primer; libraries were sequenced with Oxford Nanopore technology; and taxonomy was assigned using SILVA-based classifiers with downstream filtering for veterinary/zoonotic relevance. After quality control, 72 eukaryotic taxa were retained and DNA from at least 24 potentially pathogenic taxa was detected. *Dipylidium caninum* was most frequent (74.3%; 113/152), and opportunistic fungi/yeasts were common (e.g., *Pichia kudriavzevii* 42.4%, *Diutina catenulata* 31.5%). Zoonotic protozoa showed low-to-moderate detection frequency (*Acanthamoeba castellanii* 13.3%, *Toxoplasma gondii* 7.9%, *Balamuthia mandrillaris* 4.6%). Overall richness did not differ between fresh and dry samples (*p* > 0.05), but fresh samples contained higher richness of potentially pathogenic taxa (*p* < 0.01).

## Simple Summary

Free-roaming cats are often discussed in terms of public and environmental health, but robust baseline data are scarce in small, protected island settings. We used DNA metabarcoding (18S rRNA) to screen 152 faecal samples from community cats in La Graciosa Island (Natura 2000, Canary Islands), including fresh faeces collected from inside the traps/carriers of captured cats and dry environmental faecal deposits. We detected a high frequency of common feline parasites (*Dipylidium caninum*, about three-quarters; 74.3%) but a comparatively low detection frequency of major zoonotic protozoa (*Toxoplasma gondii*, <10%; 7.9%). Overall, these results support targeted parasite control within Trap–Neuter–Return (TNR) programmes and show that metabarcoding can provide scalable One Health surveillance, even from dry faeces.

## 1. Introduction

Unowned free-roaming domestic cats occupy a peculiar position at the interface between companion animals, urban wildlife and human societies. In many cities and rural settlements, these unowned cats are simultaneously cared for by residents and perceived as a potential threat to biodiversity and public health. Their close contact with humans, other animals and anthropogenic food sources makes them important reservoirs and potential sentinels of zoonotic infections transmitted through direct contact, vectors or environmental contamination [1,2].

Gastrointestinal parasites are a significant component of this infectious burden. Surveys of free-roaming cats from Greece, Iran, and Spain consistently report high prevalences of endoparasites, often in mixed infections, with marked geographical heterogeneity [3,4,5]. Many of these parasites have zoonotic potential and are associated with environmental contamination of public spaces, playgrounds and beaches, with implications for children, immunocompromised people and other susceptible hosts [1,4,5]. At the same time, intestinal parasites can compromise the health and welfare of cats, contributing to diarrhoea, weight loss, and subclinical morbidity that often goes unnoticed in unmanaged colonies [3,6].

Among feline parasites, *Toxoplasma gondii* stands out as a paradigmatic One Health pathogen. Felids are the only definitive hosts capable of shedding environmentally resistant oocysts, which can contaminate soil, water and food chains, with consequences for human health, livestock production and wildlife conservation [7]. Recent large-scale studies show that oocyst shedding in free-ranging domestic and wild felids is strongly modulated by human population density and climatic variables, underlining the anthropogenic drivers of transmission [8]. In coastal ecosystems, high prevalence and genotype diversity of *Toxoplasma gondii* in feral cat faeces raise particular concern for marine mammals and seabirds, which may be exposed through runoff and nearshore food webs [9]. Other parasites frequently detected in free-roaming cats, such as *Dipylidium caninum* and various nematodes and protozoa, pose a lower zoonotic risk individually but can still be relevant in highly exposed populations or under poor sanitary conditions [3,4,6].

In Europe, the European Scientific Counsel Companion Animal Parasites (ESCCAP) guidelines emphasise the need to adapt parasitological diagnosis and control strategies to local epidemiology, environmental context and cat lifestyle [6]. However, most data informing these recommendations are still based on conventional copromicroscopic techniques, which have limited sensitivity for low-intensity infections and may underestimate the diversity of protozoa, microsporidia and other eukaryotic organisms in feline faeces [6,10]. This limitation is especially relevant for free-roaming cats, whose faeces are often collected from the environment after variable periods of exposure, with unknown degrees of DNA degradation [3,5].

High-throughput DNA metabarcoding has emerged as a powerful alternative for characterising complex parasite communities [11,12]. By amplifying broad eukaryotic markers such as the 18S rRNA gene, metabarcoding can detect a wide range of helminths, protozoa, yeasts and fungi, including species that are difficult or impossible to identify morphologically [10,13,14,15]. Recent studies in wild animals and urban cats have demonstrated their potential to recover detailed profiles of pathogenic and commensal taxa from faecal samples, even in the presence of co-infections and low-abundance organisms [16,17]. At the same time, methodological challenges remain, including the dominance of host DNA in faecal extracts, amplification biases and the need for robust bioinformatic pipelines and curated reference databases [18,19,20].

Host-specific blocking primers have been proposed as an effective strategy to suppress amplification of host DNA and enhance the recovery of non-host eukaryotic sequences in mixed samples [18,21]. This approach has been successfully applied in diet studies and parasite surveys in wildlife [11]. Still, its performance in unowned free-roaming cats, and particularly in field conditions where faeces are dry or partially degraded, remains poorly documented [17,22]. Likewise, most published metabarcoding studies in cats come from continental urban settings, while insular ecosystems—often biodiversity hotspots and conservation priorities—are underrepresented despite their vulnerability to introduced predators and pathogens [2,8,9].

La Graciosa is a small (29 km^2^), inhabited island in the Canary archipelago, designated as a Natura 2000 protected area and characterised by a high density of free-roaming unowned cats closely associated with the two urban settlements. Recent work has shown that intensive Trap–Neuter–Return (TNR) campaigns on the island can achieve very high sterilisation coverage in a short time when supported by strong community engagement, highlighting both the potential and the constraints of humane management in such sensitive settings [23]. At the national level, Spain has recently adopted a comprehensive animal welfare framework that formally recognises free-roaming cats a “community cats” and promotes non-lethal management strategies [24], positioning veterinarians at the centre of One Health–oriented decision-making [25]. Within this evolving legal and social context, robust baseline data on the parasite communities and zoonotic risk associated with these community cats in protected insular environments are urgently needed to inform proportionate, evidence-based policies [2].

In this study, we use 18S rRNA metabarcoding with a cat-specific blocking primer to characterise the faecal eukaryotic parasite and pathogen community in free-roaming cats from La Graciosa. Our aims are threefold: (i) to evaluate the performance of this methodological approach under real-world field conditions, including both fresh and dry faecal samples; (ii) to describe the prevalence and diversity of parasites with potential relevance for feline health and zoonotic transmission; and (iii) to provide a baseline risk assessment for a Natura 2000 island where community cats are being managed through TNR.

## 2. Materials and Methods

### 2.1. Study Area and Study Population

**Figure 1:**
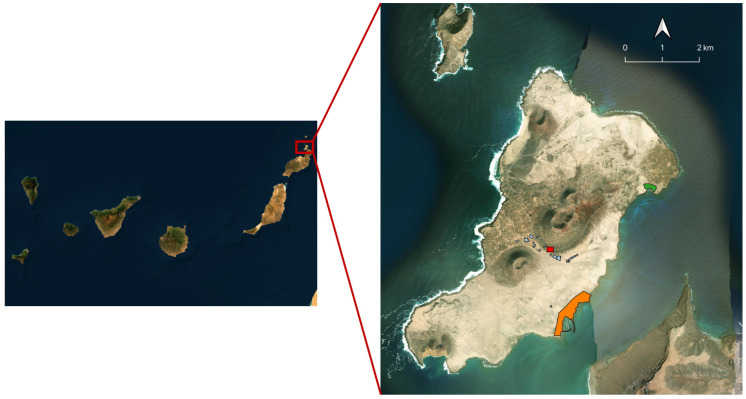
Location of La Graciosa (29.2554° N, 13.5032° W) within the Canary Islands. Colours indicate the different sampling settings where cat faeces were collected: Caleta de Sebo (orange), Pedro Barba (green), farms (light blue), and the waste dump (red). The satellite imagery was obtained from the Esri World Imagery basemap (sources: Esri, DigitalGlobe, GeoEye, i-cubed, USDA FSA, USGS, AEX, Getmapping, Aerogrid, IGN, IGP, swisstopo, and the GIS User Community).

This study was conducted on La Graciosa, designated as both a Special Area of Conservation (SAC) and a Special Protection Area (SPA) for birds within the Natura 2000 network (Figure 1). Community cats on the island are primarily concentrated around the two human settlements of Caleta de Sebo and Pedro Barba, where they rely heavily on food provided by dedicated caretakers and anthropogenic sources (e.g., garbage). Faecal collection was conducted during an intensive TNR campaign implemented in July 2024 in the urban areas of La Graciosa, as detailed in a previous study [23]. Over four consecutive days, the campaign combined a complete census of the free-roaming cat population with an intensive, high-throughput sterilisation effort. All handled cats received endo- and ectoparasite treatment, vaccination, identification, and registration as community cats—i.e., cats under municipal responsibility, in accordance with Spanish law. Individual-level demographic and health information was systematically recorded for every captured and neutered animal. In addition, to increase the overall sample size and to assess metabarcoding performance across field conditions, we collected cat faeces throughout both urban settlements and in sites within the boundaries of the protected natural area where cats are also present, including small farms, the municipal waste-disposal area, and other locations encountered during systematic walks across the island.

### 2.2. Study Design, Legal Permits and Ethical Approval

The metabarcoding survey was designed as a cross-sectional study to characterise the baseline faecal eukaryotic pathogen community of free-roaming cats in La Graciosa before large-scale antiparasitic interventions. The sampling frame included cats captured for surgical sterilisation during the TNR campaign and free-roaming cats whose droppings could be collected in the urban environment. All procedures involving animals were carried out in accordance with the legal framework established for the La Graciosa cat management programme [23].

The Environmental Council of the Cabildo of Lanzarote authorised a census and ecological study of community cats across La Graciosa (Resolution 2868/2024). A pre-sterilisation census conducted between February and May 2024 in the two main settlements of La Graciosa (Caleta de Sebo and Pedro Barba) identified 160 unowned community cats (Caleta de Sebo: 152; Pedro Barba: 8) and 24 owned cats in Caleta de Sebo (16 with outdoor access). This provides the baseline population context for the areas covered by permits in the present study. The metabarcoding dataset comprised 37 fresh faecal samples from traps/carriers of captured cats during the TNR campaign (≈23% of the estimated unowned community-cat population in the settlements) and 115 dry environmental faecal deposits collected across multiple sites (not attributable to individuals). Therefore, sampling should be considered operational/pragmatic rather than probabilistic, and environmental results are interpreted as sample-level DNA detection frequencies. Because most cats are concentrated in the urban areas of Caleta de Sebo and Pedro Barba, additional permits were issued by Teguise Municipality for activities in the urban area (reference 2024-005133) and by the Canary Islands Ports Authority for work within port facilities (reference 1168/2024). The College of Veterinarians of Las Palmas granted temporary authorisation to establish a field veterinary hospital on the island. All fieldwork and surgical procedures complied with Spanish Law 7/2023 on animal welfare and with applicable regional regulations [24,26].

In line with the authorisations obtained for the mass-sterilisation campaign in La Graciosa, ethical approval for the collection of biological samples during routine surgical procedures was granted by the Ethics Committee for Animal Experimentation at the University of Las Palmas de Gran Canaria (Resolution OEBA_ULPGC_35/2023). Ethical review and approval for this study were waived, as no experimental interventions were performed beyond standard high-volume, high-quality spay–neuter practices conducted by licenced veterinarians [27].

### 2.3. Faecal Sample Collection and Classification

A total of 152 cat faecal samples were collected in La Graciosa. Of these, 37 samples were classified as fresh and 115 as dry. All fresh samples were obtained from the interior of trapping cages or carriers belonging to cats captured in Caleta de Sebo and later admitted to the temporary veterinary facility for surgical sterilisation. Although this approach ensures cat-level traceability (one trap/carrier per animal), it does not fully exclude limited carryover of environmental eukaryotic DNA, because traps/carriers necessarily contact the sandy ground during capture and may be placed on the ground again during handling and transport. None of the cats captured in Pedro Barba defecated inside the traps, so no fresh faecal samples were available from that settlement.

The remaining 115 samples consisted of cat faecal deposits collected from locations habitually used by cats in different parts of the island. In detail, 52 dry samples were collected in Caleta de Sebo, 35 in Pedro Barba, 14 in an area of small agricultural plots located within the boundaries of the natural park, and 14 in the vicinity of the urban solid-waste compactor station (Figure 1).

The agricultural plots are used mainly by residents of Caleta de Sebo for small-scale cultivation. Permissions in this area allowed faecal sampling but did not include authorisation to trap or handle cats, so we do not have data on the individual cat responsible for the scat. The waste compactor station acts as a transfer point for municipal solid waste generated on the island, where refuse is deposited and compacted before periodic shipment to Lanzarote for treatment; there is no on-island waste treatment facility. Faeces were collected and handled with plastic gloves.

For this study, samples were categorised macroscopically as fresh faeces when they were collected directly from trapping cages shortly after deposition and showed a moist, glossy surface with no evidence of desiccation or cracking. Environmental faeces were only sampled when they still retained visible surface moisture and a slightly shiny appearance; obviously desiccated, fragmented or weathered deposits were avoided. No formal freshness score was applied beyond these macroscopic criteria. Thus, fresh samples were collected from traps/carriers and associated with captured cats; however, dry environmental deposits cannot be assigned to individual animals, and therefore repeated sampling from the same cat cannot be excluded. Accordingly, environmental results are interpreted as sample-level detection frequencies rather than population prevalence in cats.

Each sample was collected using a sterile spatula or disposable wooden stick and transferred to a labelled 15 mL Falcon tube, avoiding contact with the ground whenever possible. Collectors wore disposable gloves, which were changed between samples to minimise cross-contamination.

Because the TNR campaign in La Graciosa spanned several consecutive days, samples were stored at −20 °C on the island immediately after collection. For transport to Gran Canaria, tubes were placed in a plug-in freezer installed in the van used by the veterinary team, which remained frozen throughout the ferry crossing. Upon arrival at the laboratories of the University of Las Palmas de Gran Canaria, all samples were transferred to −20 °C freezers until DNA extraction.

### 2.4. DNA Extraction and Quality Control

DNA was extracted from 200 μL of homogenised faecal material using the Mag-Bind Stool DNA Kit (OMEGA BIO-TEK, Norcross, GA, USA), following the manufacturer’s instructions with minor adaptations to increase yield from complex matrices. For each sample, faecal material was thoroughly homogenised, and three independent extractions (triplicates) were performed. The use of triplicate extractions was intended to increase the probability of detecting low-abundance taxa and to reduce stochasticity in downstream metabarcoding results. For each extraction batch, a negative extraction control (no faecal material, kit reagents only) was included and processed in parallel through all steps. DNA was eluted in 100 μL of elution buffer and stored at −20 °C until PCR amplification.

### 2.5. 18S rRNA Metabarcoding

We targeted a short fragment of the eukaryotic 18S rRNA gene commonly used for metabarcoding of intestinal parasites and other faecal eukaryotes, in line with previously published protocols for copro-DNA analysis [10,16,28]. A 18S rRNA amplicon was generated with the newly designed primers 18S-1F (5′-CCGTCGCTACTACCGATTG-3′) and 18S-2R (5′-CTACGGAAACCTTGTTACGACT-3′). These primers included four different tags (5–6 bp) [29], for multiplexing availability, and 5′ tails (TTTCTGTTGGTGCTGATATTGC and ACTTGCCTGTCGCTCTATCTTC) for PCR barcoding with ONT barcodes.

To allow multiplexing of large numbers of samples, we adopted a combinatorial tagging strategy [26]. Using one tag per primer yields 16 primer sets for the unique identification of 16 amplicon sets. Each of these sets is tagged with 12 available ONT barcodes (EXP-PBC012, Oxford Nanopore Technologies (ONT, Oxford, UK)), enabling the final individual labelling of 192 amplicons, enough to tag each of the 152 samples analysed.

To minimise amplification of host DNA and enhance detection of non-host eukaryotes, we used a cat-specific blocking primer designed to anneal to the feline 18S rRNA template between the binding sites of the metabarcoding primers, following the host-blocking concept originally developed for mixed-template samples [18]. The blocking primer carried a 3′ C3 spacer to prevent extension and was complementary to the domestic cat (*Felis catus*) 18S rRNA sequence at the targeted locus (18S-Fcat-block: CTACCGATTGGATGGTTTAGTGAGGCCCTCGGAT/3SpC3/).

Amplification was performed in a volume of 15 μL containing GoTaq Flexi Buffer (Promega, Madison, WI, USA), 3.5 mM MgCl_2_, 200 μM of dNTP, 0.5 μM of each primer, 0.15 units of GoTaq Flexi DNA Polymerase (Promega, Madison, WI, USA), 0.2 mg/mL BSA (20 mg/mL, NEB), 5 µM blocking primer and 2 μL of faecal isolated DNA. PCR amplification protocol consisted of 95 °C for 2 min, followed by 35 cycles including 95 °C for 30 s, 58 °C for 30 s, 72 °C for 30 s, then 72 °C for 5 min, performed using a GeneAmp 9700 thermal cycler (Applied Biosystems, Foster City, CA, USA).

Reactions from each set of tag combinations (16 samples) were pooled and purified with Dr Vida MagBeads (Stabvida, Caparica, Portugal). For multiplexing, the 12 available ONT barcodes (EXP-PBC012) were incorporated into the previously purified amplicon pools via PCR barcoding. As a result, each one sample-specific amplicon was labelled by a forward and reverse tag and a dual ONT barcode. The final pool, including 170 amplicons, was purified with Gel and PCR Clean-Up (MacheryNagel, Hoerdt, France).

All PCRs included a no-template control to monitor for contamination. Amplification success, intensity, and size were verified by 2.5% agarose gel electrophoresis.

### 2.6. Library Preparation and Nanopore Sequencing

Approximately 1.25 μg of total DNA from pooled amplicons was subjected to end-repair and dA-tailing using the NEBNext Ultra II End Repair/dA-Tailing Module (New England Biolabs, Ipswich, MA, USA) and purified with magnetic beads. A total of 984 ng of pooled amplicons was sequenced with Ligation sequencing V14 (Oxford Nanopore Technologies, ONT, Oxford, UK). The 280 fmol library was loaded for sequencing in a MinION R10.4.1 flow cell (FLO-MIN114; ONT, Oxford, UK) inserted on a MinION M1kb (ONT, Oxford, UK) device connected to a portable computer, for a 5 h run.

### 2.7. Basecalling, Demultiplexing and Quality Filtering

Raw signal data were recorded in POD5 format, with basecalling, trimming and demultiplexing by ONT barcode using Dorado v0.9.5 (ONT, Oxford, UK). Additional demultiplexing by incorporated tags and filtering was performed with cutadapt v.4.10 [30]. Only reads with a size between 100 and 200 bp and a Phred quality score above 20 were retained.

### 2.8. Metabarcoding Analysis

The SILVA 138.2 SSU database was used as a source for (i) the extraction of 18S rRNA sequences delimited by the 18S-1F/18S-2R primers and (ii) the training of a Näive-Bayes classifier with the feature-classifier plugin from QIIME2 v24.10 [31]. The filtered, high-quality small reads were imported into a QIIME2 artefact submitted to the dada2 denoise plugin using ad hoc parameters. The representative sequences or amplicon sequence variants (ASVs) were submitted to taxonomic assignment by VSEARCH (identity parameter = 0.98) and Näive-Bayes methods. Additionally, significant matches generated by QIIME2 plugin were verified with NCBI BLAST.

The taxa initially recovered from cat faeces included environmental contamination, dietary components and host sequences. Taxa were retained only when supported by authoritative guidelines and/or peer-reviewed evidence of clinical relevance in cats and/or humans; all retained taxa.

### 2.9. Definition of Potentially Pathogenic Taxa and Epidemiological Analysis

For this study, selection was guided by ESCCAP guidance [6,32] and peer-reviewed syntheses on cat-associated zoonoses and feline parasitology [1,3,5,8,33]. For fungi/yeasts, we retained only taxa with published evidence of opportunistic or clinical relevance in mammals and plausible exposure pathways in cats, and we interpreted them as indicators of exposure/ecology rather than as confirmed disease agents. Zoonotic relevance was treated as a qualitative hazard level (potential public-health consequence) and not as a function of detection frequency. We classified taxa as High/Moderate/Low zoonotic relevance based on: (i) evidence of zoonotic transmission involving cats as definitive hosts/reservoirs or via cat-associated exposure pathways (e.g., environmental contamination with eggs/oocysts, bites/scratches, vectors), (ii) clinical severity and potential for irreversible outcomes in humans, and (iii) likelihood of human exposure given typical transmission routes. When taxonomic assignment could not be resolved to species level, taxa were reported at the lowest reliable rank (e.g., *Blastocystis* spp., *Malassezia* spp.). In such cases, pathogenic/zoonotic relevance was evaluated conservatively at the genus level, and no inference was made regarding specific species or viability. This approach follows published syntheses of cat-associated zoonoses and their public-health implications [1] and related zoonotic parasite frameworks in free-roaming cats [3,5].

Given the two sampling frames used in this study, we report detection metrics accordingly. For fresh samples linked to captured cats (one sample per trap/carrier), we report cat-level prevalence among captured cats as n/N. For environmental dry deposits, which cannot be attributed to individual cats, we report sample-level detection frequency as n/N and interpret it descriptively (i.e., without inferring population prevalence), acknowledging the possibility of pseudoreplication. For all proportions (prevalence in captured cats and sample-level detection frequency in environmental deposits), we computed exact binomial 95% confidence intervals using the Clopper–Pearson method, which is appropriate for low detection counts. Differences in detection frequency between sample types, sex, age class, or other covariates were explored using chi-squared or Fisher’s exact tests for categorical variables and logistic regression models for multivariable analysis. Comparisons of parasite burden between sampling sites were performed using the Kruskal–Wallis and Mann–Whitney U tests, as the underlying frequency distributions did not meet normality assumptions. All analyses were carried out with the latest available version of Jamovi (v. 2.6.45) [34]. Because one of the central aims of this study was to establish a baseline risk profile rather than to perform exhaustive modelling, quantitative analyses focused on descriptive statistics and simple comparisons, emphasising the distinction between high-detection frequency, low-pathogenicity organisms such as *Dipylidium caninum* and low-detection frequency, high-consequence agents such as *Toxoplasma gondii* and other zoonotic protozoa.

## 3. Results

### 3.1. Eukaryotic Community Composition

**Figure 2:**
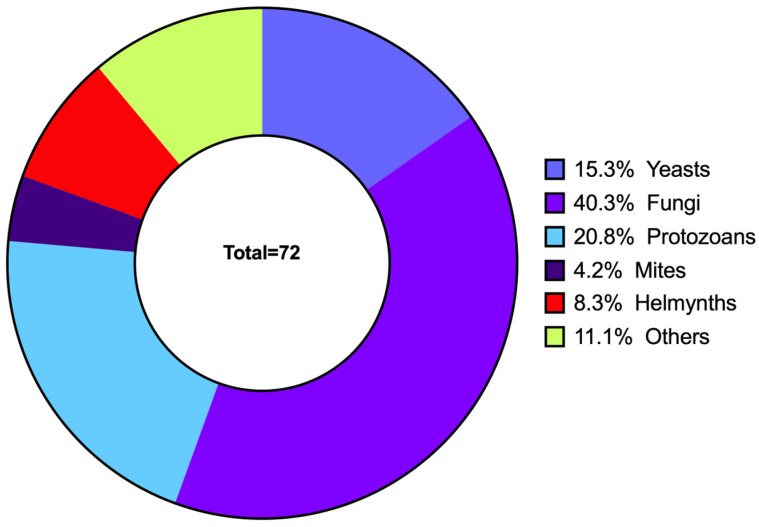
Taxonomic composition of eukaryotic organisms detected in community cat faecal samples (*n* = 72). Donut chart showing the relative abundance (percentage) of major eukaryotic groups: Yeasts (15.3%), Fungi (40.3%), Protozoa (20.8%), Mites (4.2%), Helminths (8.3%), and Others (11.1%).

After quality filtering and bioinformatic processing, a total of 72 high-quality eukaryotic ASVs reads were obtained from the 152 faecal samples. The analysis revealed a diverse community of eukaryotic taxa. Fungi were the most abundant group, accounting for 40.3% of the classified reads, followed by protozoans (20.8%), yeasts (15.3%), and helminths (8.3%). Mites and other eukaryotes constituted smaller fractions of the community (4.2% and 11.1%, respectively) (Supplementary Table S1 and Figure 2).

**Figure 3:**
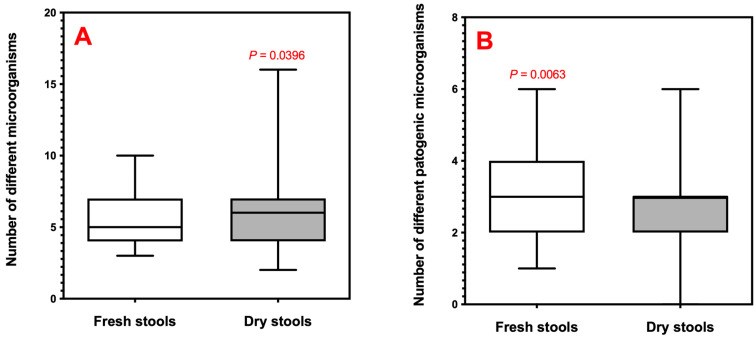
Comparison of eukaryotic diversity between fresh and dry faecal samples. (**A**) Box plot showing the number of different eukaryotic taxa detected per sample in fresh (*n* = 37) and dry (*n* = 115) samples (*p* = 0.0396). (**B**) Box plot showing the number of different potentially pathogenic eukaryotic taxa detected per sample after epidemiological filtering (*p* = 0.0063). Overall eukaryotic richness (**A**) did not differ between sample types, whereas dry samples showed a significantly lower richness of potentially pathogenic taxa (**B**).

We compared the overall microbial diversity between fresh (*n* = 37) and dry (*n* = 115) faecal samples. While the median number of different eukaryotic organisms detected per sample was slightly higher in dry stools, this difference was not statistically significant (*p* > 0.05, Mann–Whitney U test) (Figure 3A). However, fresh samples showed significantly higher pathogen richness than dry samples (*p* < 0.01, Mann–Whitney U test).

### 3.2. Detection of Potentially Pathogenic Taxa

Our 18S metabarcoding approach identified DNA from at least 24 eukaryotic taxa with known or potential pathogenic relevance for cats or humans (Table 1 and Figure 3B). Veterinary Relevance reflects the strength of evidence for clinical relevance in cats and is classified as High (well-established feline pathogen with clinically meaningful outcomes), Moderate (reported feline pathogen/opportunist with plausible clinical relevance, often context-dependent), or Low (limited evidence of pathogenicity in cats or uncertain clinical relevance). Zoonotic Relevance reflects a qualitative hazard level (potential public-health consequence and plausibility of cat-associated transmission) and is classified as High (evidence-supported zoonosis with potentially severe outcomes and/or relevant cat-associated exposure pathways), Moderate (documented zoonotic potential primarily as an opportunistic agent or with typically milder outcomes, and/or less direct cat-associated exposure pathways), or Low (limited or indirect zoonotic potential; transmission typically requires specific conditions and is not primarily driven by cat shedding).

The most frequently detected was the cestode *Dipylidium caninum*, detected in 74.3% of the samples (113/152). Several opportunistic fungi and yeasts were also widespread, including *Pichia kudriavzevii* (42.4%), *Diutina catenulata* (31.5%), and *Cladosporium herbarum* (26.1%).

Among protozoans with significant zoonotic hazard potential, *Toxoplasma gondii* DNA was detected in 12/152 samples overall samples (7.9%; exact 95% CI: 4.1–13.4). In fresh samples linked to captured cats, detection was 1/37 (2.7%; exact 95% CI: 0.1–14.2), whereas in dry environmental deposits it was 11/115 (9.6% sample-level detection frequency; exact 95% CI: 4.9–16.5). The single positive fresh sample showed a very low read count, and therefore this finding is interpreted conservatively as a low-frequency signal in individually traceable samples. The free-living amoeba *Balamuthia mandrillaris*, a rare but highly pathogenic agent, was detected in 4.6% of samples (7/152). Other protozoa, such as *Acanthamoeba castellanii* and *Blastocystis* spp., were present at detection frequencies of 13.3% and 3.9%, respectively. The cat-specific coccidian *Cystoisospora felis* was detected at a low detection frequency of 0.7% (1/152).

### 3.3. Geographic Distribution and Co-Occurrence Patterns

The detection frequency of several eukaryotic organisms varied significantly across the four sampling sites (Farms, Caleta de Sebo, Pedro Barba, Garbage dump) (Table 2).

The Kruskal–Wallis test revealed significant geographic structuring for eight taxa (*p* < 0.05) (Table 3).

**Figure 4:**
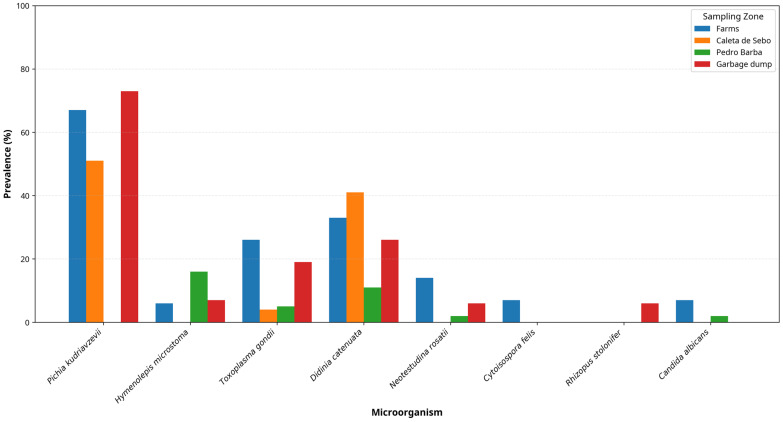
Detection frequency of the 8 eukaryotic organisms with statistically significant geographic variation (Kruskal–Wallis, *p* < 0.05) across the four sampling settings. Bars represent the percentage of positive samples for each eukaryotic organism in each sampling site (Farms, Caleta de Sebo, Pedro Barba, Garbage dump). Data visualisation was performed using Python (Python 3.11.0rc1) with the matplotlib library.

Across sampling sites, taxa showing significant heterogeneity (Kruskal–Wallis, *p* < 0.05) displayed distinct distributional patterns (Table 2; Figure 4). *Toxoplasma gondii* was most frequently detected in Farms (26.7%) and the Garbage dump (20.0%), whereas detection frequencies were lower in Caleta de Sebo (4.2%) and Pedro Barba (5.1%). *Pichia kudriavzevii* showed consistently high detection frequencies in three sites—Garbage dump (73.3%), Farms (66.7%) and Caleta de Sebo (51.6%)—but was not detected in Pedro Barba (0.0%). *Hymenolepis microstoma* was most frequently detected in Pedro Barba (15.4%), with lower detection frequencies in Farms (6.7%) and the Garbage dump (6.7%), and it was not detected in Caleta de Sebo (0.0%). These site-level contrasts should be interpreted cautiously because the survey is cross-sectional and detection frequencies may vary over time; additionally, environmental deposits cannot be attributed to individuals and are therefore interpreted as sample-level detection frequencies rather than population prevalence (Figure 4).

Co-occurrence analysis identified several significant positive and negative associations between taxa (Table 4).

**Figure 5:**
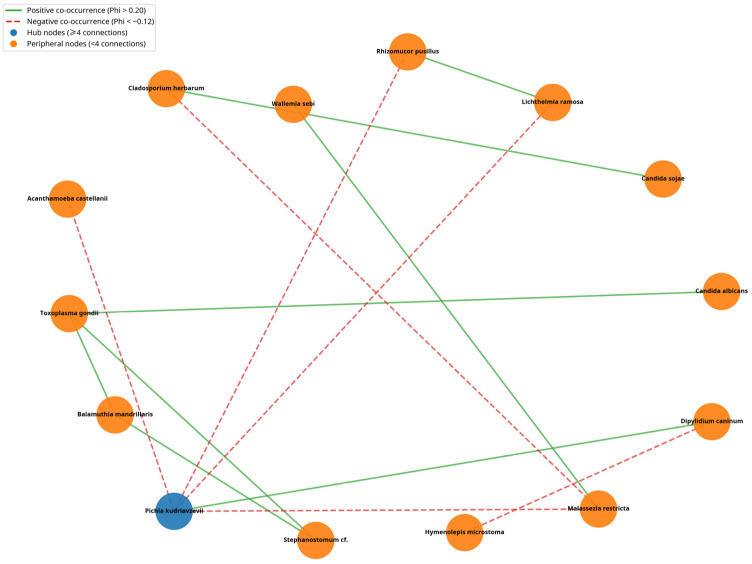
Co-occurrence network of eukaryotic organisms detected in community cat faeces. Nodes represent taxa; edges represent statistically supported pairwise associations based on the Phi coefficient computed on presence/absence data. Positive associations (Phi > 0.20) indicate co-occurrence, whereas negative associations (Phi < −0.12) indicate negative association (mutual exclusion/non-co-occurrence). Hub nodes are defined as taxa with ≥4 significant connections. Network construction and visualisation were performed in Python using NetworkX (v 3.6.1).

A strong positive co-occurrence was observed between *Balamuthia mandrillaris* and *Toxoplasma gondii* (Phi = 0.342). *Toxoplasma gondii* also co-occurred with *Stephanostomum* spp. and *Candida albicans*. The network analysis highlighted *Pichia kudriavzevii* as the main hub node (≥4 significant connections), whereas *Toxoplasma gondii* showed three significant associations under the applied thresholds (Figure 5; Table 4).

## 4. Discussion

This study provides the first comprehensive baseline of eukaryotic pathogens in a community cat population from a protected Natura 2000 site using 18S rRNA metabarcoding. Our results in this setting challenge the prevailing narrative of community cats as vectors of high zoonotic risk, instead revealing a parasite community characterised by low detection frequency of high-risk zoonotic pathogens when compared to global epidemiological data. These findings likely support the viability of TNR as a One Health strategy that reconciles animal welfare, public health protection, and wildlife conservation objectives.

### 4.1. Methodological Approach and Parasite Community Characterisation

The use of 18S rRNA metabarcoding with a cat-specific blocking primer proved effective for characterising complex parasite communities in faecal samples collected under real-world field conditions. This approach successfully detected helminths, protozoa, fungi, yeasts, and even mites in a single multiplex reaction, demonstrating advantages over traditional microscopy, which typically requires separate procedures for each parasite group [10]. The recovery of DNA from rare or low-abundance organisms, such as *Balamuthia mandrillaris*, underscores the sensitivity advantage of this method over conventional coproscopy [11,12].

A notable finding was the significantly higher richness of pathogenic taxa in fresh samples compared to dry samples (Figure 3B), despite no difference in overall microbial richness. One plausible explanation is differential DNA integrity in environmental deposits: exposure time, desiccation, UV and inhibitors can reduce detectability, particularly for low-template targets [35]. This is consistent with the broader molecular scatology literature showing that highly degraded scats often yield lower-quality DNA and may reduce detection sensitivity for some taxa [18,35]. Therefore, differences between fresh and dry samples should be interpreted primarily as differences in detection rather than definitive absence/presence [10]. For future surveillance programmes, this indicates that fresh samples are preferable for accurate assessment of pathogen burden, although environmental sampling remains valuable for community characterisation and cost-effective large-scale surveys [36,37].

The co-occurrence network analysis (Figure 5, Table 4) indicates a structured pattern of eukaryotic detections, with *Pichia kudriavzevii* emerging as a highly connected taxon. Strong positive associations between specific taxa (e.g., *Balamuthia mandrillaris* and *Toxoplasma gondii*; Phi = 0.342) may reflect shared exposure routes, correlated environmental conditions, or other common drivers of co-detection, rather than direct biological interaction. From a management perspective, these descriptive patterns can help prioritise surveillance and control efforts by focusing on highly connected taxa and the sets of organisms that tend to be detected together [38].

However, we should note that the sampling faced limitations and inference. Our sampling comprised two frames: (i) fresh faeces collected from traps/carriers of captured cats during TNR, and (ii) dry environmental deposits collected across the island. While the former can be interpreted at the level of captured cats, environmental deposits cannot be attributed to individuals; therefore, repeated sampling from the same cat cannot be excluded. Consequently, metrics derived from environmental deposits are reported and discussed as sample-level detection frequencies, not as population prevalence, and inference to the whole cat population is limited.

If the same individual contributes more than one environmental deposit, detection frequencies may overestimate true prevalence in cats, particularly for taxa with sustained shedding or when sampling is concentrated in hotspots frequented by a subset of individuals. Conversely, if sampling over-represents individuals that are consistently uninfected, detection frequencies could be biassed downward for some taxa. Importantly, non-independence would also lead to underestimated uncertainty (overly narrow confidence intervals) and potentially inflated type I error in inferential comparisons that assume independent samples. For these reasons, we interpret results from environmental deposits descriptively and avoid population-level prevalence claims based on this sampling frame.

### 4.2. Contextualising Zoonotic Risk: Global Epidemiology and La Graciosa Baseline

Cross-study comparisons are provided for contextualisation and should be interpreted cautiously due to differences in sampling frames (captured-cat faeces vs. environmental deposits), endpoints (DNA detection vs. oocyst shedding or serology), analytical pipelines, and local ecological contexts; therefore, we avoid formal between-study inference.

The detection of *Toxoplasma gondii* DNA in 12/152 samples overall (7.9%; exact 95% CI: 4.15–13.38) provides a low baseline signal within the available literature. However, this result needs to be interpreted cautiously given differences in sampling frames and endpoints across studies (DNA detection vs. serology or oocyst shedding). Importantly, when stratified by sample type, cat-linked fresh samples showed only 1/37 positives (2.7%; exact 95% CI: 0.07–14.16), whereas dry environmental deposits showed 11/115 positives (9.6%; exact 95% CI: 4.87–16.47). Because dry deposits cannot be attributed to individual cats and may include repeated contributions from the same animals, we treat the dry-sample estimate as sample-level detection frequency rather than population prevalence. The single positive fresh sample also had a very low read count, supporting a conservative interpretation of low cat-linked detection in this baseline survey.

These results are lower than pooled global serology-based estimates reported for domestic cats, although direct comparison with pooled oocyst shedding estimates is limited because endpoints differ (DNA detection vs. shedding/viability) [8,39]. Serology-based studies report global seroprevalence of *Toxoplasma gondii* in domestic cats at approximately 35%, with regional studies reporting rates ranging from 30% to 40%, whereas the prevalence of oocyst shedding in feline faeces has been estimated globally at 2.6% [8,40]. In our study, the cat-linked fresh-sample detection was low (1/37; 2.7%), while the overall estimate (12/152; 7.9%) is driven mainly by dry deposits.

Geographic heterogeneity is also well documented. A meta-analysis of *Toxoplasma gondii* seroprevalence in domestic cats worldwide revealed striking geographic variation, with seroprevalence ranging from less than 10% in Thailand, Taiwan, and Angola to over 70% in Qatar and Ethiopia [39]. Similarly, seroprevalence rates in domestic cats were high in Australia (52%) and Africa (51%), whereas the lowest seroprevalence (27%) was reported from Asia. These global patterns reflect the influence of climate, prey availability, and human population density on *Toxoplasma gondii* transmission.

More directly relevant to our study, a recent investigation of feral cat populations in coastal California reported *Toxoplasma gondii* DNA detection in 25.9% of faecal samples (94/362; 95% CI: 21.7–30.7), which is 3 times higher than La Graciosa (12/152; 7.9%; 95% CI: 4.1–13.4) [33].

The spatial clustering of *Toxoplasma gondii* in the Farms and Garbage dump areas (Figure 4) is epidemiologically informative. This pattern likely reflects the presence of more abundant intermediate hosts (rodents, birds) in semi-rural agricultural or resource-rich habitats, creating local hotspots of transmission. Importantly, this geographic heterogeneity demonstrates that not all areas of La Graciosa pose equal risk, a key insight for targeted public health interventions. The strong co-occurrence of *Toxoplasma gondii* with *Balamuthia mandrillaris* in these high-risk sites (Phi = 0.342) may reflect shared environmental risk factors—such as proximity to soil and water sources—rather than a true epidemiological association [40,41].

While *Toxoplasma gondii* is undoubtedly a significant zoonotic pathogen, the 7.9% detection frequency in La Graciosa must be weighed against contextual factors and exposure pathways. Basic hygiene measures (handwashing and avoiding contact with cat faeces/contaminated soil) are effective at reducing transmission, and DNA detection does not equate to clinical disease risk unless viable oocyst production, environmental persistence, and human exposure align. A broader synthesis on *Toxoplasma gondii* in domestic and wild felids emphasises that public-health relevance depends critically on local epidemiological context rather than the mere presence of infected felids [7].

Overall, the available evidence from La Graciosa does not support an overly alarmist framing of free-roaming cats as an immediate, island-wide zoonotic threat in this setting [7,9]. Future [8] work combining faecal molecular screening with serology and, where feasible, viability-oriented approaches would allow a more direct assessment of public-health relevance.

The detection of *Dipylidium caninum* DNA in 74.5% of samples (113/152) is the most striking finding of our study. However, this high detection frequency must be correctly interpreted. Published prevalence rates for *Dipylidium caninum* in cats range from 1.8% to 52.7%, with higher rates consistently reported in free-roaming, unmanaged cat populations [3]. Although direct quantitative comparison is limited by differences in sampling design, diagnostic approach, and endpoints, our finding of 74.5% is above the upper end of the reported range but entirely consistent with the lifestyle and environmental exposure of community cats on La Graciosa. The epidemiological significance of *Dipylidium caninum* is fundamentally different from that of *Toxoplasma gondii*. While technically a zoonosis, *Dipylidium caninum* transmission to humans requires accidental ingestion of infected flea intermediate hosts—an event that is rare, preventable, and typically results in mild gastrointestinal symptoms [42]. The high prevalence of *Dipylidium caninum* DNA is therefore more indicative of a high flea burden in the cat population than of a high public health threat. This finding has direct implications for animal welfare: flea-infested cats suffer from pruritus, anaemia, and secondary infections, particularly in young or immunocompromised individuals. The high detection frequency underscores the urgent need for integrated ectoparasite and endoparasite control in the TNR programme, but it does not justify alarmist public health messaging [42].

The detection of *Balamuthia mandrillaris* DNA in 4.8% of samples (7/152) is, to our knowledge, one of the first reports of this free-living amoeba in domestic cat faeces. *B. mandrillaris* is the causative agent of granulomatous amoebic encephalitis (GAE), a rare but severe disease in humans and other mammals [43]. The presence of its DNA in an environment frequented by humans demands careful attention. However, several important epidemiological and clinical caveats must be noted. First, it is unclear whether this DNA represents true intestinal infection, passive passage after ingestion of contaminated soil or water, or external contamination of the faecal sample. *B. mandrillaris* is a free-living amoeba ubiquitous in soil and water environments worldwide; its presence in cat faeces may simply reflect environmental exposure rather than a unique risk posed by cats. This caveat also applies to ‘fresh’ samples, which were collected from deposits inside traps/carriers rather than rectal swabs, and therefore may still contain residual environmental DNA introduced via trap/carrier contact with sandy soil. Second, while *B. mandrillaris* infection in humans is invariably severe—with an estimated case fatality rate exceeding 90% in documented cases—the actual incidence of human infection is extraordinarily rare. A comprehensive epidemiological review of *B. mandrillaris* disease in the United States from 1974 to 2016 identified only 101 confirmed cases over a 42-year period in a population of over 300 million, indicating an incidence of approximately 0.008 cases per million per year [43]. The disease predominantly affects immunocompromised individuals, with documented risk factors including HIV/AIDS, organ transplantation, and chronic use of immunosuppressive drugs. Among the 101 documented U.S. cases, only 10% (10 patients) survived their infection, and the median survival time from symptom onset to diagnosis was several months, reflecting the severity of the disease but also its rarity and the difficulty of ante-mortem diagnosis. Third, the actual transmission risk from cats to humans remains poorly characterised, and to our knowledge no role has been attributed to cats as vectors of this disease, and the unique reference assigning a role of animals as vectors is related to tropical forest human-primate interactions [44].

Rather than interpreting this finding as evidence of cats as dangerous vectors, we propose that it highlights the need for further research on the role of cats in the environmental cycle of *B. mandrillaris* and the actual magnitude of human health risk. The presence of *B. mandrillaris* DNA in cat faeces may reflect passive environmental contamination or transient carriage but does not establish cats as a significant epidemiological source. In the interim, standard precautions (glove use during handling of cat faeces, hand hygiene) are sufficient to mitigate any theoretical risk, as these measures are effective against environmental exposure to free-living amoebae. This finding should not be weaponized to justify lethal control measures but rather should motivate rigorous epidemiological investigation into the actual role of cats in *B. mandrillaris* transmission ecology. Accordingly, detections of free-living environmental taxa should be interpreted conservatively as DNA signals consistent with exposure and/or environmental carryover, not as evidence of infection or shedding.

A significant finding of our study is the high detection frequency and diversity of opportunistic fungi and yeasts, particularly *Pichia kudriavzevii* (42.4%), *Diutina catenulata* (31.5%), and *Cladosporium herbarum* (26.1%). These organisms are often overlooked in traditional parasitological surveys, yet they provide valuable epidemiological insights. While their direct pathogenicity in cats is likely low, they can represent opportunistic threats in severely immunocompromised humans [10]. The striking geographic pattern—with *Pichia kudriavzevii* reaching 73.3% detection frequency at the garbage dump—strongly suggests a link to fermenting organic waste and anthropogenic food subsidies. This observation exemplifies a key One Health principle: the feline microbiota is shaped not by intrinsic pathogenicity but by environmental and anthropogenic factors [45,46]. The high detection frequency of these fungi at the waste facility highlights the importance of improved waste management as a public health intervention, independent of cat control measures. However, an equally important insight emerges from the broader framework of anthropogenic resource provisioning and wildlife-pathogen dynamics. Recent meta-analyses demonstrate that the provision of controlled, high-quality food resources to wildlife populations can substantially reduce infection loads by improving host nutritional status and immune function, while simultaneously reducing the behavioural incentive to consume contaminated environmental food sources [45,46]. Applied to community cats, this principle suggests that the provision of complete commercial cat food (formulated to meet nutritional requirements) represents a concrete One Health intervention that operates through multiple pathways: (1) improved nutritional status and immune competence, reducing susceptibility to opportunistic infections; (2) reduced reliance on fermented garbage and waste, thereby decreasing exposure to environmental pathogens including fungi, bacteria, and parasites; and (3) reduced attraction to the waste facility itself, lowering population-level exposure to contaminated food sources.

By reducing the availability of fermenting organic matter through improved waste management and simultaneously providing controlled food provisioning as part of TNR programmes, authorities could simultaneously reduce the prevalence of these opportunistic organisms, decrease the attractiveness of the garbage dump as a food source for cats, and improve the overall health and immune competence of the community cat population. This integrated approach—combining waste management with food provisioning—represents a practical, evidence-based One Health intervention that addresses both the proximate (garbage dump) and ultimate (nutritional deficiency) drivers of high fungal prevalence in community cats.

### 4.3. TNR as an Evidence-Based One Health Strategy

Our findings provide robust evidence supporting TNR as a viable One Health strategy for managing community cat populations. The low-to-moderate DNA detection frequency of high-risk zoonotic pathogens in La Graciosa—particularly when contextualised against global epidemiological baselines—demonstrates that the public health argument against community cats does not apply in such context, and likely in EU regions where high standards of public health systems exist.

TNR improves the health and quality of life of community cats by preventing uncontrolled reproduction, reducing intraspecific aggression, and enabling targeted veterinary interventions (vaccination, antiparasitic treatment) [47,48]. This aligns with evolving legal frameworks such as Spain’s Law 7/2023 on animal welfare [24,26], which explicitly recognises community cats and promotes non-lethal management strategies [2]. When combined with selective antiparasitic and antimicrobial interventions, TNR can reduce the prevalence of zoonotic pathogens without resorting to lethal control. The low baseline detection frequency of high-risk pathogens in La Graciosa indicates that the public health burden is manageable through evidence-based veterinary care. The integration of One Health and One Welfare principles in TNR programmes ensures that animal welfare improvements are not achieved at the expense of public health [2,5,25,49].

By stabilising and consequently reduce cat populations through fertility control TNR avoids the welfare costs of culling while still addressing the ecological impact of free-roaming cats in the long-term [50]. For small, isolated populations like those on La Graciosa, TNR combined with habitat management and prey protection measures offers a more sustainable approach than lethal control [23,50,51,52]. Recent evidence demonstrates that TNR is effective in reducing cat colony size over time while improving animal health outcomes, and public support for TNR is documented to be high in many communities, reflecting a growing recognition that humane management strategies are both ethically justified and epidemiologically sound [53,54].

### 4.4. Future Research and Implications for Management

This study provides a crucial baseline for La Graciosa prior to large-scale antiparasitic and vaccination campaigns. A key limitation is that we have not yet evaluated the impact of these interventions on parasite burden. Future research should assess the post-intervention detection frequency through repeat metabarcoding surveys at 3-, 6-, and 12 months post-TNR to quantify the reduction in parasite DNA following antiparasitic treatment. Comparative studies should evaluate the effectiveness of targeted treatment (e.g., anti-coccidial drugs only for cats in high-risk sites) versus universal broad-spectrum antiparasitic protocols. The impact of vaccination (e.g., against *Toxoplasma gondii*) on seroconversion and DNA shedding should be assessed [55]. Additionally, it would be valuable to assess whether TNR-mediated changes in cat population structure (age, sex, reproductive status) influence parasite epidemiology, and to measure the persistence of parasite DNA in the environment following TNR implementation, to understand the timeline for risk reduction [56].

These investigations will transform La Graciosa into a model system for evidence-based, One Health-oriented community cat management in protected areas. The parasite community of La Graciosa’s free-roaming cats is characterised by low detection frequency of high-risk zoonotic pathogens when compared to global epidemiological baselines. The most frequently detected parasite, *Dipylidium caninum*, poses minimal public health risk despite its high detection frequency. *Toxoplasma gondii*, while epidemiologically important, occurs at a detection frequency well below global averages. The detection of *Balamuthia mandrillaris* is novel and warrants further investigation but should not be interpreted as evidence of cats as exceptional vectors of this rare pathogen.

These findings support the conclusion that TNR, combined with selective veterinary interventions, is a scientifically justified, ethically defensible, and epidemiologically sound strategy for managing community cats in La Graciosa. The narrative that free-roaming cats represent an unmanageable zoonotic threat is not supported by the evidence from this Natura 2000 site. Instead, our data demonstrate that proportionate, evidence-based management can simultaneously improve animal welfare, protect public health, and maintain the ecological integrity of protected areas. The integration of institutional governance frameworks with epidemiological baselines, as demonstrated in recent work on TNR implementation in insular landscapes [57], underscores the importance of coordinated, multi-level policy approaches that align animal welfare, public health, and conservation objectives.

### 4.5. Limitations of the Study

This study has several limitations that should be considered when interpreting the findings. First, the survey is cross-sectional and provides a baseline snapshot; therefore, it cannot capture temporal variability in detection frequencies, which may fluctuate over time and season. Second, the sampling frame included (i) fresh faeces collected from traps/carriers linked to individual captured cats and (ii) dry environmental deposits that cannot be attributed to individuals; consequently, dry-sample results are interpreted as sample-level detection frequencies rather than population prevalence, and repeated contributions from the same cats cannot be excluded. Third, although strict field handling procedures and laboratory negative controls were applied, limited environmental carryover (e.g., from sandy soil in traps/carriers) and low-level contamination cannot be fully excluded. Finally, metabarcoding provides DNA detection rather than direct evidence of parasite viability, infectivity, or active shedding, and comparisons with studies based on serology or oocyst shedding should be made cautiously.

## 5. Conclusions

This study shows that 18S rRNA metabarcoding combined with a host-blocking primer is a robust tool to characterise eukaryotic taxa of veterinary and zoonotic interest in free-roaming cat faeces, including challenging field matrices combining cat-linked fresh samples and dry environmental deposits. The approach recovered a diverse assemblage of helminths, protozoa, fungi and yeasts, and revealed spatial heterogeneity that is unlikely to be captured by conventional coproscopy alone.

In La Graciosa, baseline detection patterns were dominated by common feline parasites (notably *Dipylidium caninum*), whereas major zoonotic protozoa showed comparatively low DNA detection, and signals were strongly shaped by sampling frame (cat-linked fresh samples versus non-attributable environmental deposits). Overall, the findings support integrating systematic parasite (and vector) control within Trap–Neuter–Return as a pragmatic One Health measure, coupled with targeted management actions (e.g., waste control and appropriate feeding practices). The baseline presented here provides a reference for post-intervention surveillance and is transferable to other protected insular contexts where proportionate, evidence-based decisions are required.

## Figures and Tables

**Table 1:** Pathogenic taxa detected in free-roaming cats and their indicative relevance. Includes detection frequency (%), number of positive samples, veterinary pathogen relevance (High/Moderate/Low), and zoonotic relevance (High/Moderate/Low) for each taxon identified.

Taxon	Type of Eukaryotic Organism	Cats’ Pathogen	Human Pathogen (Zoonosis)	Detection Frequency (%)	95% CI	Veterinary Relevance	Zoonotic Relevance ^a^
*Dipylidium caninum*	Cestode	X	X	74.3	66.6–81.1	High	Low
*Pichia kudriavzevii*	Yeast		X	42.4	34.2–50.4	Low	Moderate
*Diutina catenulata*	Fungus		X	31.5	24.3–39.6	Low	Moderate
*Cladosporium herbarum*	Fungus		X	26.1	19.5–34.1	Low	Moderate
*Acanthamoeba castellanii*	Protozoan		X	13.3	8.2–19.6	Low	Moderate
*Malassezia restricta*	Fungus		X	12.7	7.7–18.8	Low	Moderate
*Toxoplasma gondii*	Protozoan	X	X	7.9	4.1–13.4	Moderate	High
*Candida sojiae*	Yeast	X	X	7.3	3.7–12.6	Moderate	Moderate
*Balamuthia mandrillaris*	Protozoan	X	X	4.6	1.9–9.3	Moderate	High
*Hymenolepis microstoma*	Helminth	X	X	4.8	1.9–9.3	Moderate	Low
*Blastocystis* spp.	Protozoan	X	X	3.9	1.5–8.4	Moderate	Low
*Lichtheimia ramosa*	Fungus	X	X	4.2	1.5–8.4	Moderate	Low
*Malasseziomyces* spp.	Fungus	X	X	2.6	0.7–6.6	Moderate	Low
*Rhizopus stolonifer*	Fungus	X	X	0.6	0.0–3.6	Moderate	Low
*Candida albicans*	Yeast	X	X	0.6	0.0–3.6	Moderate	Low
*Candida glabrata*	Yeast	X	X	0.6	0.0–3.6	Moderate	Low
*Rhizomucor pusillus*	Fungus		X	5.3	2.3–10.1	Low	Low
*Debaryomyces fabryi*	Yeast		X	3.9	1.5–8.4	Low	Low
*Demodex brevis*	Mite		X	3.3	1.1–7.5	Low	Low
*Neotestudina rosatii*	Fungus		X	2.4	0.7–6.6	Low	Low
*Wallemia sebi*	Fungus		X	1.3	0.2–4.7	Low	Low
*Trichosporon coremiiforme*	Fungus		X	1.3	0.2–4.7	Low	Low
*Stephanostomum* spp.	Helminth		X	0.6	0.0–3.6	Low	Low
*Cystoisospora felis*	Protozoan	X		0.7	0.0–3.6	Low	Low
*Trichosporon*	Fungus		X	0.6	0.0–3.6	Low	Low

^a^ Zoonotic relevance indicates qualitative hazard level (public-health consequence and plausibility of cat-associated transmission) and is reported independently of detection frequency/prevalence; it is not a quantitative risk estimate.

**Table 2:** Detection frequency (%) of eukaryotic organisms detected in community cat faecal samples across four sampling sites in La Graciosa. Rows represent individual eukaryotic organisms; columns represent sampling sites. Data presented as a percentage of positive samples within each sampling site. Includes all taxa detected with detection frequency ≥ 0.6%.

Eukaryotic Organism	Farms	Caleta de Sebo	Pedro Barba	Garbage Dump
*Dipylidium caninum*	66.7	81.1	64.1	66.7
*Acanthamoeba castellanii*	0.0	15.8	15.4	6.7
*Stephanostomum* cf.	0.0	0.0	2.6	0.0
*Diutina catenulata*	33.3	41.1	10.3	26.7
*Wallemia sebi*	0.0	1.1	2.6	0.0
*Cystoisospora felis*	6.7	0.0	0.0	0.0
*Rhizomucor pusillus*	0.0	2.1	10.3	13.3
*Debaryomyces fabryi*	0.0	6.3	0.0	0.0
*Neotestudina rosatii*	13.3	0.0	2.6	6.7
*Cladosporium herbarum*	26.7	30.5	20.5	13.3
*Pichia kudriavzevii*	66.7	51.6	0.0	73.3
*Malassezia restricta*	6.7	11.6	17.9	13.3
*Demodex brevis*	0.0	3.2	5.1	0.0
*Trichosporon coremiiforme*	0.0	2.1	0.0	0.0
*Toxoplasma gondii*	26.7	4.2	5.1	20.0
*Rhizopus stolonifer*	0.0	0.0	0.0	6.7
*Balamuthia mandrillaris*	0.0	2.1	7.7	0.0
*Hymenolepis microstoma*	6.7	0.0	15.4	6.7
*uncultured fungus*	0.0	10.5	25.6	0.0
*uncultured Basidiomycota*	0.0	4.2	0.0	0.0
*Lichtheimia ramosa*	0.0	5.3	0.0	13.3
*Candida sojae*	13.3	10.5	0.0	0.0
*Candida albicans*	6.7	0.0	0.0	0.0
*Candida glabrata*	0.0	0.0	2.6	0.0

**Table 3:** Eukaryotic organisms with statistically significant variation in detection frequencies across sampling sites, based on the Kruskal–Wallis test (*p* < 0.05). Includes chi-square (χ^2^) values, *p*-values, effect size (ε^2^), and the sampling site with the highest detection frequency for each taxon. Effect sizes are interpreted as small (ε^2^ < 0.06), medium (0.06 ≤ ε^2^ < 0.14), or large (ε^2^ ≥ 0.14).

Eukaryotic Organism	χ^2^	*p*-Value	ε^2^ (Effect Size)	Sampling Site with the Highest Observed Detection Frequency ^a^
*Pichia kudriavzevii*	41.150	<0.001	0.252	Garbage dump (73.3%)
*Hymenolepis microstoma*	14.269	0.003	0.088	Pedro Barba (15.4%)
*Toxoplasma gondii*	12.354	0.006	0.076	Farms (26.7%)
*Diutina catenulata*	12.238	0.007	0.075	Caleta de Sebo (41.1%)
*Neotestudina rosatii*	10.919	0.012	0.067	Farms (13.3%)
*Cystoisospora felis*	9.933	0.019	0.061	Farms (6.7%)
*Rhizopus stolonifer*	9.933	0.019	0.061	Garbage dump (6.7%)
*Candida albicans*	9.933	0.019	0.061	Farms (6.7%)

^a^ This column is descriptive only and does not imply significant pairwise differences between sites; the Kruskal–Wallis test assesses overall heterogeneity across the four sites.

**Table 4:** Top significant co-occurrence (positive) and mutual exclusion (negative) patterns between eukaryotic organisms, based on the Phi coefficient. (A) Top 10 positive co-occurrences (Phi > 0.20), suggesting potential co-occurrence patterns that may reflect shared exposures or ecological drivers. (B) Top 10 negative co-occurrences (Phi < −0.12), suggesting mutual exclusion (non-co-occurrence). Phi coefficient ranges from −1 (perfect negative association) to +1 (perfect positive association).

Eukaryotic Organism A ^a^	Eukaryotic Organism B ^a^	Phi Coefficient
A. Positive Co-occurrences (Top 8)		
*Balamuthia mandrillaris* [P]	*Stephanostomum* spp. [H]	0.442
*Lichtheimia ramose* [F]	*Rhizomucor pusillus* [F]	0.347
*Balamuthia mandrillaris* [P]	*Toxoplasma gondii* [P]	0.342
*Malassezia restricta* [Y]	*Wallemia sebi* [F]	0.290
*Stephanostomum* spp. [H]	*Toxoplasma gondii* [P]	0.267
*Candida albicans* [Y]	*Toxoplasma gondii* [P]	0.267
*Candida sojae* [Y]	*Cladosporium herbarum* [F]	0.259
*Dipylidium caninum* [H]	*Pichia kudriavzevii* [Y]	0.248
B. Negative Co-occurrences (Top 6)		
*Lichtheimia ramose* [F]	*Pichia kudriavzevii* [Y]	−0.181
*Malassezia restricta* [Y]	*Pichia kudriavzevii* [Y]	−0.181
*Pichia kudriavzevii* [Y]	*Rhizomucor pusillus* [F]	−0.152
*Cladosporium herbarum* [F]	*Malassezia restricta* [Y]	−0.144
*Dipylidium caninum* [H]	*Hymenolepis microstoma* [H]	−0.127
*Acanthamoeba castellanii* [P]	*Pichia kudriavzevii* [Y]	−0.120

^a^ Abbreviations for organism groups: Y, yeasts; F, filamentous fungi; P, protozoa/free-living amoebae; H, helminths.

## Data Availability

The original contributions presented in this study are included in the article/Supplementary Materials. Further inquiries can be directed to the corresponding author.

## Supplementary Materials

The following supporting information can be downloaded at: https://www.mdpi.com/article/10.3390/ani16030431/s1, Table S1: Microbial taxa identified in feline faecal samples from La Graciosa, with detection frequencies in fresh and dry samples and statistical significance of differences.
