# Supplementary material for: Low Zoonotic Pathogen Burden in Free-Roaming Cats Revealed by 18S rRNA Metabarcoding: A Baseline Study from an Insular Natura 2000 Site in Spain
Source: Animals (Basel). 2026 Jan 29;16(3):431. doi: 10.3390/ani16030431 (PMC12896981; doi:10.3390/ani16030431)
Supplement: Supplementary file 1 [file animals-16-00431-s001.zip › animals-4082227-supplementary.pdf]

Supplementary Table S1. Microbial taxa identified in feline faecal samples from La Graciosa, with detection frequencies in fresh and dry samples and statistical significance of differences.

Taxon	Type of microorganism	Detection frequency (fresh faeces) (%)	Detection frequency (dry faeces) (%)	p-value
Acanthamoeba (Acanthamoeba castellanii)	Protozoan (amoeba)	8,1	15	
Acari (Demodex brevis)	Mite	0	3,9	
Acari (Myobia musculi)	Mite	8,1	6,3	
Acari (Puccinia striiformis)	Mite	2,7	8,7	
Aspergillus (Aspergillus versicolor)	Fungi	43,2	18,9	0,0048
Aspergillus (uncultured Ascomycota)	Fungi	5,4	0	0,0498
Aureobasidium (Aureobasidium iranianum)	Yeast	0	0,8	
Azygiida (Otodistomum cestoides)	Plathyhelminth	0	5,5	
Badhamia (Physaridae spp.)	Fungi	0	0,8	
Balamuthia (Balamuthia mandrillaris)	Protozoan (amoeba)	2,7	3,1	
Blastocystis (unidentified guinea)	Protozoan	0	5,5	
Candida (Candida albicans)	Yeast	0	0,8	
Candida (Candida sojae)	Yeast	16,2	4,7	0,0451
Candida (Diutina catenulata)	Fungi	45,9	27,6	
Cephalobaenida (Raillietiella spp.)	Arthropod (pentastomid)	2,7	4,7	
Cladosporium (Cladosporium herbarum)	Fungi	27	26	
Cladosporium (Zasmidium cellare)	Fungi	0	1,6	
Conidiocarpus (Conidiocarpus caucasicus)	Fungi	0	0,8	
Cyclophyllidea (Dipylidium caninum)	Cestode	91,9	69,3	0,005
Cyclophyllidea (Hymenolepis microstoma)	Cestode	0	6,3	
Cystoisospora (Cystoisospora felis)	Protozoan (coccidian)	0	0,8	
Cystoisospora (Cystoisospora suis)	Protozoan (coccidian)	0	2,4	
Debaryomyces (Debaryomyces fabryi)	Yeast	13,5	0	0,0005
Didymosphaeriaceae (Paraphaeosphaeria sporulosa)	Fungi	8,1	1,6	
Eimeriorina (Toxoplasma gondii)	Protozoan	2,7	9,4	
Eugregarinorida (Stylocephalus giganteus)	Protozoan	0	0,8	
Eugregarinorida (Xiphocephalus triplogemmatus)	Protozoan (apicomplexa)	16,2	20,5	
Filobasidium (Filobasidium floriforme)	Fungi	2,7	31,5	0,0002
Filobasidium (Filobasidium magnum)	Fungi	0	0,8	
Geotrichum (Galactomyces candidus)	Fungi	8,1	21,3	
Hanseniaspora (Hanseniaspora valbyensis)	Yeast	0	7,9	
Haplotaxida (Enchytraeus spp.)	Metazoan (annelid worm)	0	0,8	
Herpotrichia (Herpotrichia parasitica)	Fungi	0	1,6	
Heterobranchia (Cavolinia uncinata)	Metazoan (marine planktonic)	10,8	4,7	
Kazachstania-candida (Kazachstania exigua)	Yeast	2,7	8,7	
Leptomyxa (Ripidomyxa spp.)	Protozoan (amoeba)	0	0,8	
Lichtheimia (Lichtheimia ramosa)	Fungi	0	5,5	
Malassezia (Malassezia restricta)	Fungi	2,7	15,7	0,0474
Melanospora (Melanospora asymmetrica)	Fungi	0	6,3	
Mycamoeba (uncultured freshwater)	Protozoan (amoeba)	0	0,8	

Naganishia (Naganishia vishniacii)	Fungi	13,5	26,8	
Nakaseomyces-candida (Candida glabrata)	Yeast	0	0,8	
Neotestudina (Neotestudina rosatii)	Fungi	0	3,1	
Oikopluridae (Oikopleura spp.)	Metazoan (marine planktonic)	0	6,3	
Opisthorchiida (Stephanostomum spp.)	Trematode	0	0,8	
Penicillium (Penicillium chrysogenum)	Fungi	0	5,5	
Penicillium (Penicillium expansum)	Fungi	13,5	2,4	0,0151
Phalansterium (Phalansterium spp.)	Protozoan (amoeba)	0	1,6	
Pichia (Pichia kudriavzevii)	Yeast	62,2	37	0,0113
Pichia (Pichia membranifaciens)	Yeast	13,5	11	
Rhizomucor (Rhizomucor pusillus)	Mold	0	6,3	
Rhizopus (Rhizopus stolonifer)	Fungi	0	0,8	
Saccharomyces (Saccharomyces cerevisiae)	Yeast	16,2	20,5	
Salpida (Thalia democratica)	Metazoan (marine planktonic)	0	13,4	0,0138
Solicoccozyma (Solicoccozyma terreus)	Fungi	2,7	7,9	
Spiromyces (Spiromyces aspiralis)	Fungi	0	3,1	
Spongillida (Baikalospongia bacillifera)	Fungi	2,7	0	
Starmerella-candida (Candida etchellsii)	Yeast	0	1,6	
Strigeidida (Psettarium nolani)	Trematode	2,7	5,5	
Stygamoebida (Stygamoeba regulata)	Protozoan (amoeba)	0	3,1	
Tetraphyllidea (Spiniloculus spp.)	Cestode	43,2	46,5	
Trichosporon (Trichosporon coremiiforme)	Fungi	5,4	0	0,0498
Unclassified Appendicularia (Oikopleuridae)	Metazoan (marine planktonic)	0	0,8	
Unclassified Basidiomycota (Malasseziomycetes spp.)	Fungi	10,8	0	0,0023
Unclassified Discosea (Dactylopodida)	Protozoan (amoeba)	0	0,8	
Unclassified Malasseziaceae (Malassezia spp.)	Fungi	21,6	9,4	
Unclassified Thecofilosea	Protozoan	10,8	14,2	
Unclassified Trebouxiophyceae (Trebouxiophyceae)	Green microalga (*Chlorophyta*)	8,1	3,1	
Unclassified Tremellomycetes (Trichosporon)	Fungi	0	0,8	
Unclassified Tremellomycetes (Trichosporonaceae spp.)	Fungi	2,7	0	
Unclassified Tubullinea (Echinamoebida)	Protozoan (amoeba)	0	4,7	
Wallemia (Wallemia sebi)	Fungi	0	1,6	
